# Reflections on alma mater: Origin and evolution of Ruhr University Bochum

**DOI:** 10.12669/pjms.40.8.10111

**Published:** 2024-09

**Authors:** HR Ahmad, Marianne Schläfke

**Affiliations:** 1 HR Ahmad, Dept. of Biological and Biomedical Sciences, The Aga Khan University, Karachi, Pakistan; 2 Marianne Schläfke, Ruhr University Bochum, 4630 Bochum, Germany

The parliament of the State of North Rhein Westphalia in 1961 passed the bill to establish a university being christened as Ruhr Universität Bochum, Germany. Between 1964 and 1970 the university faculty of arts and literature took the lead followed by science while thirteen state of the art buildings were constructed at the periphery of city Bochum upon the river Ruhr. Along with this construction work during these years, a group of scientists led by founding fathers from biological and medical faculties under the guidance of HH Loeschke, Professor of Physiology, Faculty of Medicine and Johann Schwarzkopff, Professor of Biology, Faculty of Biological Sciences planned, programmed, and put to work an institution of a special *research programme project* known as “Sonderforschungsbereich” [SFB]. The scientist team moved into provisional laboratories in a building at the Fredericka Strasse in Bochum. Here the experimental work of some doctoral candidates started, who became later university professors in Germany. Thus, the Fredericka – Street is celebrated as the primordial birthplace of scientists before the commissioning of Ruhr University Bochum in 1970.

The first SFB was commissioned to life sciences as a programme project “*Reception and Processing of Biological Signals: Basic Research and Application*” at RUB in 1970. Johann Schwarzkopff was the first head of SFB 114. He described the vibrant economical era in Bochum during the late sixties, the foundation, and the programme of SFB and its role in establishment of research in life sciences at the Ruhr Universität Bochum.

The first SFB was commissioned to faculty of medicine in physiological sciences as a programme project “*Chemical Signals and the Control of Breathing and Circulation*” under HH Loeschke and his team of scientists. Loeschke’s team explored the central feedback loop of the respiratory control system starting with Marian Schläfke as his first MD student at Ruhr University. Integrated topics were:

- Organization of Chemical Respiratory Drives
- Organization of Central Respiratory and Circulatory Rhythm Generators,
- Acid Base Homeostasis of Brain ECF
- Pathophysiological Phenomena: Sudden Infant Death Syndrome, Sleep apnea, and Bronchomotor disorders.

The work of HH Loeschke’s group on the integrative aspects of the control system, has been lucidly presented on his farewell symposium at Ruhr University Bochum.^1^ SFB 114 was financially supported by German Research Council from 1972 to 1986. These fifteen years of published research work has been treasured in a book on “Biological Signal Processing”.^2^ The major areas of research were cellular and multicellular analysis of signal processing. Each part starts with an overview that describes the general progress in the corresponding area during the last 15 years. It is followed by articles in which notable results are described in more detail.

As to the content of this report in the form of a published book, the reader might judge for self how far the ambitious aims have been achieved to accomplish internationally recognized research within a few years of foundation of Ruhr University Bochum. How scientists and research infrastructure intercepted to nurture faculty in research-based education for public service. However, SFB 114 officially ended in 1986 after well supported programme projects by German Research Council. This conclusion was associated with the retirement of the founders of SFB. Professor HH Loeschke and Professor J Schwarzkopff set a good example to pass on the baton to the next generation of scientists. Therefore, a thorough reorganization of cooperation in life sciences research was sought.

Since SFB 114 dealt with a rather general programme project with scientists from different faculties, a new pathway from general to specific was sought. The new “Research Unit” focused on “*Membrane Control of Cellular Activity”* and was successfully funded by German Research Council. This programme project included the molecular analysis of the chain of signaling events at the cell membrane leading to a specific cell response. The cells to be investigated were in skeletal muscle, heart, photoreceptor, and ciliate.

Interestingly, impulses for organizing a second biological research group came from scientists of the second generation to investigate cognition based on neuronal circuits in the brain of man and higher organisms. It was anticipated that results of the *neuronal networking of cognition* would model a new generation of computer.

Another interesting theme of research was the investigation of *neuronal mechanisms in sensory guided orientation of behavior in vertebrates*. The investigations included specific nerve cell function as well as the cerebral representation of space and movement for goal directed behavior. Central themes from neurophysiology were investigations of structure-function relationships for specific performance of neurons controlling orientation and direction specificity in the visual cortex.

RUB was founded at a timeframe when economy was experiencing Wirtschaftswunder under Chancellor Ludwig Eberhardt in 1961. Its success was based on high quality research mentors adequately endowed faculty with modern equipment, infrastructure and cycles of grants by German Research Council.

The situation changed for the second-generation faculty due to severe economic crisis in Ruhr district. Every crisis opens a new door for adaptation. Therefore, great efforts are required to convince politicians and public forums that substantial investments were needed to make Ruhr University Bochum attractive for nurturing a new generation of creative scientists for the nation. Eventually, this strategy will turn out to be the best investment for North Rhein Westphalia to respond to new challenges of modern technology.

Dedicated to the fond memories of founding mentors of research HH Loeschke, Faculty of Medicine and J Schwarz Kopff, Faculty of Biology to have laid the foundation of Ruhr University Bochum and showed us the pathway how to integrate basic biological concepts into medicine for public service. ME Schläfke and HR Ahmad are disciples of HH Loeschke and are indebted to pass on his baton of way of doing in depth research with emphasis on preserving the intellect and integrity of the next generation.
